# Gaining the courage to see and accept oneself: Group-based compassion-focussed therapy as experienced by adolescent girls

**DOI:** 10.1177/1359104520931583

**Published:** 2020-06-06

**Authors:** Anna Bratt, Ing-Marie Gralberg, Idor Svensson, Marie Rusner

**Affiliations:** 1 Department of Psychology, Faculty of Health and Life Sciences, Linnaeus University, Sweden; 2 Child & Adolescent Psychiatric Clinic, Södra Älvsborg Hospital, Region Västra Götaland, Sweden; 3 Institute of Health and Care Sciences, Sahlgrenska Academy, University of Gothenburg, Sweden; 4 Department of Research, Region Västra Götaland, Södra Älvsborg Hospital, Sweden

**Keywords:** Compassion-focussed therapy, adolescent psychiatry, adolescent mental health problems, group therapy, qualitative research

## Abstract

Shame and self-stigmatisation are common in adolescents with mental health problems, and can hinder their recovery. Compassion-focussed therapy (CFT) help people address challenging experiences and emotions with courage, wisdom, and care. However, no previous studies have examined whether CFT is helpful for adolescents with mental health problems. The present study aimed to describe lived experiences regarding group-based CFT based on the perspectives of a sample of adolescent girls who were recruited from a child and adolescent psychiatric outpatient clinic in Sweden. In-depth interviews were conducted with six girls, aged 15 to 17, using a reflective lifeworld research approach. The results showed that participating in group-based CFT means *gaining the courage to see and accept oneself through meeting with peers who are experiencing similar difficulties*. When sharing experiences in a group, new perspectives and an acknowledgement that mental and emotional struggle are normal arise, and a sense of inner peace and belonging emerges. Instead of hiding from society, it is possible to participate in everyday life, ask for help, and asserting oneself. CFT can provide a promising method for empowering young people with mental health problems, helping them feel connected with others, and fostering in them the strength to show their true personalities.

## Introduction

Young people with mental health problems (i.e. diagnosable mental-health conditions) represent a vulnerable group with unique needs. Besides the suffering caused by their mental health problems, such people can have difficulties attending and achieving in school, participating in leisure activities, and spending time with peers (Anttila et al., 2015). Further, instead of becoming more independent, young people with mental health problems often become more dependent on their parents (Hasson-Ohayon et al., 2014). It is also common for adolescents with mental health problems to internalise public stereotypes, prejudice, and discrimination; that is, to self-stigmatise (Kaushik et al., 2016). In turn, such shame and self-criticism can contribute to, and maintain, mental health problems (Gilbert & Procter, 2006; Leaviss & Uttley, 2015), particularly in adolescence (Castilho et al., 2017; Cunha et al., 2012; Gilbert & Irons, 2009; Marsh et al., 2018). A special focus in compassion-focussed therapy (CFT) is helping clients regard themselves in a caring and supportive manner, rather than criticising and blaming themselves (Gilbert, 2010, 2017). However, whether this approach is helpful for adolescents with mental health problems requires further exploration.

Psychotherapy research mainly focusses on treatment effectiveness, using quantitative measures of symptom change and functioning; as a result, the perspectives of young participants, which represent qualitative data, are somewhat neglected (Krause et al., 2019). Overall, few qualitative studies have explored participants’ experiences of psychotherapy (Binder et al., 2012). However, as CFT is a novel intervention with one of the first studies published in 2006 by Gilbert and Procter, participants’ perspectives regarding whether CFT is relevant and/or helpful are important (Clapton et al., 2018; Lawrence & Lee, 2014).

CFT is a multi-modal therapy model based on evolutionary psychology, attachment theory, cognitive behavioural theory (CBT), and neuroscience (Gilbert & Procter, 2006). Although the number of studies exploring the effectiveness of CFT have increased substantially in recent years (Beaumont & Hollins, 2015; Kirby & Gilbert, 2017; Leaviss & Uttley, 2015), studies that explore stand-alone CFT (i.e. without a combination of treatment-as-usual or CBT) are limited. The potential power of CFT for adolescents and their parents have been outlined (Carona et al., 2017), but needs to be explored in a clinical setting.

In conclusion, adolescents who are living with mental health problems experience challenging emotions and difficulties attending and achieving in school, and can also be excluded from friend and leisure-time activities. Further, feelings of worthlessness, shame, self-blame, and self-stigmatisation can hinder recovery and worsen any existing sense of sadness, loneliness, and anxiety. However, no previous studies have examined whether CFT is helpful for adolescents with mental health problems. Therefore, the aim of this qualitative study was to describe the lived experiences of group-based CFT from the perspective of participating adolescent girls.

## Methods

### Design

For the present research, the ‘reflective lifeworld’ approach was chosen to obtain and describe lived experiences of group-based CFT from adolescent girls’ perspectives. Reflective lifeworld research (RLR), which is based on phenomenology and continental philosophy, focusses on lived experiences from an insider perspective (Dahlberg & Dahlberg, 2019; Dahlberg et al., 2008). The core methodological principles of RLR are ‘openness’, ‘flexibility’, and ‘bridling’ (Dahlberg et al., 2008); this means that researchers should endeavour to maintain an open mind towards participants’ accounts, without hastily developing conclusions; be flexible when exploring the data; and ‘bridle’ their natural tendency ‘to seek to determine how things actually are’.

### Context

This study was conducted at a child and adolescent psychiatric outpatient clinic in western Sweden. The clinic specialises in treating complex mental health difficulties in children and adolescents aged 3 to 18 years; psychiatric treatment is provided free of charge for all patients (Swedish Healthcare, 2019).

### Intervention

The group-based CFT programme applied in this research was developed and adapted by the first author for use with both adolescents with mental health problems and their parents; this was based on the transdiagnostic CFT approach (Gilbert, 2009, 2010). The programme consisted of eight 2-hr group sessions, in accordance with the principles of CFT, and these were adapted to the needs of the participants. For example some participants needed more concrete and visual information. Parallel group sessions were simultaneously conducted for the adolescents and their parents. The present study focused on adolescents’ experiences; parental experiences have been described in a previous study (Bratt et al., 2019).

For an overview of the content of each session, see Table 1. The focus of the first session was to allow the participants to familiarise themselves with the other group members, and to create a safe group environment. The group leaders did not know the reasons the group members were receiving psychiatric care (their diagnoses) or why they had chosen to join the group. The group members explained their problems in their own words, as well as their hopes or worries regarding joining the group. One focus topic was learning about the motivational basis of emotions, and how our brain function differently based on the three circles model of our affect-regulation systems: threat, drive and soothing/safeness. The group members were introduced to their compassionate self, and were taught how to develop this inner side of themselves. Another consistent theme was learning how to understand the role of their self-critique side of themselves. As part of the CFT, the participants engaged in mindfulness- and compassion-based exercises (e.g. soothing breathing, creating a safe place and chair-work). Chair-work is a psychotherapeutic technique where the participants sit on different chairs, where each chair represents different emotions or parts of oneself (Bell et al., 2019). One chair represents the compassionate self, and the adolescent can have a dialog with the other emotions/parts of herself. Additionally, the adolescent girls were encouraged complete their homework assignments with their parents; examples of such homework assignments include soothing rhythmic breathing, mindfulness and compassion-based exercises.

**Table 1.** Summary of the content of the group-based adolescent CFT intervention.

Session number and title	Content and key elements	Specific practice(s)
1. Welcome – why we are here.	Getting to know each other, learning how to create a feeling of safety in the group, and exploring the three circles model.	Drawing your own three circles. Reflecting, together with the group members, why you think this drawing appears in this way, and what you need to improve your balance. *Soothing breathing* exercise
2. Recognising, exploring, and understanding our emotions.	How do you recognise the different feelings you experience? What do you think and how do you behave depending on your feeling/feelings?	Writing, drawing, and discussing our *multiple selves. Calming and soothing stone mindfullness* exercise
3. Our kind and wise inner self.	Exploring the difference between our compassionate self and our self-critical self.	*Safe space visualization* exercise *Compassionate self- visualization* practice.
4. Taking care of yourself in difficult moments.	Exploring the function of our self-critical element. Becoming compassionate toward ourselves when experiencing difficulties.	*STOP* exercise. Describe a difficult situation. What happened, and how did you act? Stop, breath, and think, what would your kind and wise self recommend you to do? *Hand on heart meditation.*
5. Listening to yourself and others.	Describing a difficult situation to a group member, and listening to another member’s experience. What would your compassionate self recommend you to do in such a situation? What compassionate advice do the other group members have?	Listening exercise. Formulation, what do you need, what do you strive for? Performing a body scan and finding a safe place in the body.
6. First-aid kit for difficult moments.	Creating a step-by-step emergency plan for difficult moments.	Multiple selves and chair-work. Describe a difficult situation. What happened, and how did you act? What did you feel? Select the most difficult emotions. Based on the story of the difficult situation, the other group-members act the selected emotions. Making a compassion bracelet.
7. Effecting change through small steps.	What do you need when you suffer? What do we all need when we suffer? Compassionate tips for yourself and others.	Compassionate letter writing. Writing short compassionate tips for yourself and the other group members.
8. Who are you, and who do you want to be?	Review of the content. What are my values? If you are your kind and wise version of yourself, where do you want to go? What is important for you?	

### Ethical considerations

The Regional Ethical Review Board in Gothenburg, Sweden, granted ethical approval for this study (No. 330-16). The confidentiality of the data and protection of the participants’ identities were ensured. The adolescents received oral and written information regarding the study before they agreed to participate in the CFT intervention. Specifically, they were given a description of the study aim and procedure, their right to withdraw at any time, and how their privacy would be maintained. Further, the researchers’ contact details were provided, and the participants were informed that participation in the study would not affect their psychiatric treatment and that any information they provided during the research would not be added to their psychiatric journal. All participants, as well as their parents, then provided written consent regarding participation and use of their data.

### Procedure

All adolescents in the psychiatric outpatient clinic who satisfied the inclusion criteria (see below; *n* = 203) were asked if they would like to participate in group-based CFT, and the parents of consenting adolescents were then asked to join the parental group. Ultimately, 30 adolescents agreed to join the CFT intervention. Their eligibility was determined by the unit’s treatment team at recruitment. Specifically, the inclusion criteria were: (a) aged 14 to 17 years, and (b) receiving treatment at the clinic. Meanwhile, the exclusion criteria were: (a) a history of acute suicidality or trauma, acute psychotic symptoms, serious self-harm, substance misuse, and/or autism, and (b) unable to speak Swedish at a level sufficient to engage in this study. The CFT was performed by either the first author, who was a clinical psychologist and psychotherapist, or a social worker who specialised in child psychiatric care. Both of these group leaders had completed CFT training with the Compassionate Mind Foundation in the United Kingdom. For each group, an assistant was present during the group sessions; these assistants had extensive experience in psychiatry and other related fields, but no specific training in CFT.

### Interview participants

All adolescents (*n* = 19; 17 girls) who completed the group-based CFT programme were invited to participate in an interview; ultimately, six girls, aged 15 to 17, agreed. The reasons the others declined to participate were not collected. Table 2 presents information concerning the interviewees and the girls’ own descriptions of their problems and, where participants disclosed it, their diagnoses.

**Table 2.** Interviewees’ characteristics.

Interview no.	Participant	Parents’ marital status/participated in parental group (yes/no)	Number of sessions attended	Description of interviewee’s problems
1	17-year-old girl	Married/Yes	8	Anxiety, depression, difficulties with peer/social relationships.
2	16-year-old girl	Married/No, participated in only one parental group meeting	7	School-related anxiety, depression and stress.
3	17-year-old girl	Married/Yes	8	Anxiety, stomach pain, difficulties attending school, suspected ADHD and dyslexia.
4	17-year-old girl	Married/No, participated in only one parental group meeting	6	Anxiety, depression and stress as a result of school and family situations.
5	17-year-old girl	Married/Yes, mother	6	Depression, anxiety, procrastination, difficulties attending school.
6	15-year-old girl	Divorced/Yes	6	School-related anxiety, loneliness and stress.

ADHD: attention deficit hyperactivity disorder.

### Data collection

Participant recruitment proceeded continuously from January 2017 to October 2018; in total, five CFT groups were created, with a minimum number of 3–4 adolescents in each group. Interviews were held from August 2017 to December 2018. A clinical psychotherapist (second author), who did not participate in the CFT intervention, conducted all face-to-face interviews at the child and adolescent psychiatric clinic, a setting chosen by the participants. Information about the interviewer, and the goals of the research had been given to the participants by their group-leader. The interviews lasted for 31 to 60 min (median = 48.55), and were recorded digitally and consequently transcribed verbatim. The adolescent girls’ lived experiences regarding the group-based CFT, from their own perspectives, were the focus of the open, in-depth interviews. According to the RLR approach, the interviews should represent an open dialogue between the interviewer and interviewee, and should not be based on a formal set of questions. Thus, all interviews began with the following open-ended question: ‘I am interested in your experience of participating in the CFT intervention. Can you tell me about it’? The interviewer encouraged the participants to reflect on their experiences by posing questions such as, ‘Can you tell me more about that?’ and ‘Can you give an example of your experience?’ This approach to interviewing is intentional within RLR, where the focus is on the interviewee’s perspective on the phenomenon.

### Data analysis

The first and third authors analysed the transcribed data in accordance with the RLR approach described by Dahlberg et al. (2008). The interview transcripts were read several times to obtain a sense of the overall meaning. Meaning units were then extracted, and units with similar meanings were grouped together in clusters. The essential structures of the meaning units were found by flexibly switching perspectives between the entire transcript and its constituent parts until the meaning structure emerged. The essence, or the essential meaning, represents the core of a phenomenon; all unique and different aspects of the data, (i.e. the variations), represent the constituents.

## Results

Through the analysis process described above, we found that the essential meaning of participating in group-based CFT is *gaining the courage to see and accept oneself through meeting with peers who are experiencing similar difficulties*. When sharing experiences in a group, new perspectives arise. There is no reason to feel ashamed, as all members are essentially the same. Acknowledging that experiencing difficulties is normal brings inner peace and a sense of belonging. Instead of withdrawing from society, it is possible to participate in everyday life, ask for help, and set boundaries, if needed.

The following three constituents further explicate the meaning of the phenomenon: (a) a sense of clarity and relief; (b) feeling connected; and (c) asserting oneself.

### A sense of clarity and relief

The lived experience of participating in the group-based CFT intervention meant finding inner calm through practicing different breathing and visualisation exercises. Trying to concentrate was difficult when feeling restless and having trouble sitting still. At times, it was not possible to practice between sessions, but at other times, such as stressful situations, it was helpful to use soothing breathing. This phenomenon meant acquiring an understanding of why people feel distressed, why difficult emotions affect people’s ways of thinking and acting, and what people can do to help relieve their suffering. The phenomenon also involved a realisation that all humans share the same methods of negotiating and addressing emotional problems. Before acquiring this knowledge, difficult feelings were mixed together in a chaotic manner. By learning to create small sub-goals and to focus on one thing at a time, a small change and a degree of stress relief was experienced. Through practical exercises such as chair-work, new perspectives arise, such as seeing one’s own and others’ situations from an external perspective.

*Sometimes you get scared of your feelings; when you get angry when you don’t want to, or when you think you shouldn’t be angry. However, when someone else is sitting there and telling you why she would be angry if she experienced that same situation, it feels somewhat okay to be angry, even if you still think you should not be angry. It is good to know that I am only human.*

Caring about oneself, rather than trying to avoid difficulties, was a new experience. One girl recognised that understanding herself was not the same thing as feeling pity. She mentioned that, when she felt self-pity, she did not have the strength to get out of bed. However, caring for herself gave her the strength to do difficult things such as go to school.

Shame and self-criticism were decreased through several different practices such as writing a compassionate letter to oneself, and writing compassionate notes to each other. There was a sense of relief when acknowledging that other people might not be judgemental, even though that is how it feels like when feeling ashamed.

### Feeling connected

Participating in group-based CFT intervention was initially difficult. However, levels of nervousness decreased when realising that the other group members were ordinary girls of the same age. Trusting can be difficult; for instance, there was a fear that other people, outside the group, would find out about the girls feelings. Some girls needed a relatively long time to feel safe, and one girl never felt completely safe. For this latter girl, unfamiliarity with the other group members and experiencing difficulties answering questions and expressing herself made it hard for her to fully integrate into the group. She, nevertheless, had a strong desire to develop the connectedness the others seemed to have.

At the beginning of the intervention, talking about problems was difficult. As they were accustomed to hiding their feelings, sharing these feelings represented both a relief and a somewhat odd experience. The therapy leaders helped the participants to regard the group as a safe place, where discussing difficulties felt normal and mundane. Eventually, sharing experiences felt pleasant, and senses of shame and loneliness decreased. Recognising that many people feel distress, not only those in the group, made the girls feel safer and calmer.

*I always believed that I was the only one who felt like this. Now, I feel better, because I no longer think that I am the only one who has these emotions.*

A sense of being listened to, seen, understood, and acknowledged arose when discussing difficult things in the group. For some, this was a new experience. When leaving the group at the end of the sessions one could feel energised and happy.

*Meeting others who are also suffering, and who, because they seem okay and look fine, you would not think are suffering, has made a great difference. I think that if other people feel bad, then there is nothing wrong with me just because I suffer, so I can talk about it, too. Admitting to others that I suffer was like admitting it to myself, and we were able to talk and understand each other.*

For some, feeling connected and not being alone was the most important benefit of participating in the group. Recognising that others shared the same experiences was comforting. Some girls mentioned that the other girls in the group seemed to benefit more from the group than they did; meanwhile, others mentioned that the group discussions represented the first times they really felt understood. Hearing about others’ experiences meant to be able to understand the perspectives of others, and could also gain an understanding of themselves.

### Asserting oneself

The lived experience of group-based CFT meant developing a new way of relating to oneself. Instead of criticising oneself for having mental health problems, it was more acceptable to show feelings to others. There was no reason to be ashamed, and this resulted in changes in school, among friends, and at home. It became easier to ask for help, and there was a recognition that other people could be available if needed.

*I guess I have the courage to admit when things are messed up. For example, when I feel that I am beginning to become anxious in school, or when it gets too much and I get so tired. . . Then I can say to a friend, ‘I do not feel okay at all right now’. Instead of trying to hide my feelings. . . I talk to someone, or go to a teacher to get help, or I can call my parents; things I was not able to do before. These things used to be really difficult.*

The phenomenon entailed a gradual increase in courage, through which it became possible to set and communicate their limits to friends, parents, and teachers when feeling mistreated. There was an experience of being able to participate and a feeling of being involved, rather than isolation. However, the experience of becoming stronger and developing the ability to stand up for oneself had both good and bad consequences. One girl described feeling lonelier as a result.

*I used to think it was my fault if I was mistreated by friends, but now I have set boundaries, and I am not going to hang out with those who treat me badly. However, all of my old friends treated me badly, so now I do not have anyone, except my boyfriend, to hang out with. . .*

For some girls, school attendance improved. Meanwhile, one girl mentioned that, although she continued to be frequently absent, she now enjoyed school when she was able to attend. Before the CFT, she had always found it difficult to attend school, but now, on days she was unable to attend, she actually desired to be in school.

Some girls described that when their parents received help in the parental group, they felt better. They could now talk to their parents and receive support without a sense of guilt. It was as if the parents had developed a better understanding of the girls’ situation, as they (the parents) no longer asked lots of questions or harangued the girls about their mental health problems. It was easier to accept oneself when parents were calmer.

*It is really nice. I used to feel guilty about feeling bad. At times when I was not able to go to school, she [the girl’s mother] was very upset. Once, she started to cry because she was so worried. It was my fault and I felt so guilty. However, now I think she has begun to understand. It is as if we can walk straight [be proud] together. I do not feel guilty anymore, and it is easier to be honest when I struggle.*

Some parents did not attend the parental group, and the daughters attended the CFT groups by themselves. One girl said that this situation did not bother her, as her parent regularly tried to listen to her, and gave her support at home. However, not having a parent in the parental group could also be a disappointment. Those whose parents participated in the parental group seemed to feel that they were understood, and there was a desire for an opportunity to share the content with parents.

## Discussion

In this study, the essential meaning of participating in group-based CFT was *gaining the courage to see and accept oneself through meeting with peers who are experiencing similar difficulties*. This phenomenon was further explicated by its three constituents 1) a sense of clarity and relief; 2) feeling connected; and 3) asserting oneself.

Feeling connected and not being alone were an essential part of the phenomenon. Hearing the other group members discuss their difficulties induced a process of recognising and accepting oneself. Shame and guilt decreased, and it was easier to ask for help if needed. Adolescence is a period of integration; specifically, the integration of all the different aspects of who we are to form our identity and ourselves (Siegel, 2015). We form our identity in the context of our relationships with others. To describe this, Siegel (2015) presented the term ‘MWe’, which means ‘a “me” and a “we”’. In the integrating process, we are linked to others, in a ‘we’ that belongs to a larger whole, ‘a we-defined self’ (p. 301, 2015). The CFT seemed to enable the girls to feel more connected to each other and to their parents. Having parallel groups for the adolescents and their parents enabled both parties to connect to each other in a new way. Bratt et al. (2019) had similar findings in their parental study, where parents described the CFT intervention as enabling them to share their feelings and enhance their ability to communicate with their children. Both the group context and parallel groups for parents seemed to allow adolescents to experience greater compassion toward themselves, as well as to accept compassion from their parents or peers. Compassion occurs in relation to others and can be divided into three types: self-to-other, other-to-self and self-to-self (Gilbert, 2017). Compassion from others and self-compassion (self-to-self) in particular seem to reduce shame and self-criticism, and, in turn, mental health problems such as depression (Hermanto et al., 2016; Kirby et al., 2019). However, these processes can also trigger grief, specifically grief related to care and affection that had been missing in the past (Gilbert, 2010). This underlines that parallel groups are important when conducting CFT; if adolescents are beginning to discuss their feelings and are desiring connection but have no one to connect with, the process can represent a painful experience.

The girls mentioned experiencing a sense of relief when they realised that they were like everyone else, that they could share their difficulties with others, and that they could set boundaries when they were treated unfairly by people. These results are very promising, as adolescents who have mental health problems often have a sense of being excluded, feel that they are different to their peers, and have thoughts that they might never recover from their mental health problems (Anttila et al., 2015). In addition, Anttila et al. (2015) found that adolescents who have mental illness hope psychiatric treatment will help them feel more included in peer groups, gain more independence from their parents, and allow them to live a typical adolescent life, with less mental pain (Anttila et al., 2015). The results of the present study showed that a couple of girls experienced becoming able to participate in school and in friend-related activities. Another experience was becoming stronger and being able to assert oneself, and setting boundaries. However, not allowing others to treat you badly could also mean becoming lonelier. Compassionate behaviour takes different forms, and does not only concern soothing yourself or being nice to yourself. Asserting oneself is not easy and requires courage, as does self-compassion and engaging and acting on suffering (Gilbert, 2017).

Understanding and accepting oneself was intertwined with the process of understanding others. When the girls felt safe and a sense of belonging, they were able to engage in everyday-life activities. CFT may be helpful for empowering young people who have mental illness. Empowerment is the opposite to self-stigmatisation, and includes a sense of being ‘normal’, being able to discuss mental health problems without negative labelling, and being able to share and discuss one’s own condition (Kranke et al., 2015).

This study contributes to the development of a deeper understanding of how CFT is experienced by adolescent girls who have mental illness. Overall, in child and adolescent psychotherapy research, the children’s and adolescents’ own perspectives have not been considered (Krause et al., 2019); therefore, the present study provides data regarding a previously unexplored phenomenon. Moreover, qualitative research studies are scarce in this field, and associated quantitative studies rarely include measures such as personal growth, interpersonal relationships, school performance, motivation, and calmness (Krause et al., 2019). Furthermore, studies in this area have traditionally focussed on single conditions, such as depression or anxiety disorders, and there is a lack of studies that include clinical samples where comorbidity (the co-occurrence of two or more mental-health disorders) is common (Gergov et al., 2015; Riosa et al., 2011).

## Limitations

As a result of the small sample size in this study, the variation and richness of the content and the transferability of the results may be limited. Of the 19 adolescents who completed the CFT intervention, 17 were girls, and only girls agreed to join the interview study. Therefore, the lived experience of group CFT might differ for boys. The reasons for boys’ non-participation is not known, however, it might be due to gender-related stigma about self-disclosure. Moreover, the adolescent girls’ descriptions of the phenomenon are set in a Swedish context, and may not be applicable to other cultural contexts. However, the variation within our sample seems to be sufficiently rich to provide important insight regarding how adolescent girls experience and perceive CFT.

The researchers used different strategies to ensure objectivity in the data analysis and the validity of the findings. Although intrusion of the researchers’ biases was inevitable, we continuously endeavoured to minimise this as far as possible. When analysing the data for the inherent meanings, to prevent premature decisions regarding the findings the first and third author reviewed and compared their analyses.

## Conclusion

After participating in group-based CFT, the adolescent girls gained the confidence and courage to develop self-acceptance. Shame and thoughts of being different decreased after meeting the other group members. Despite these promising findings, however, further studies are needed to explore whether CFT can help empower young people who have mental health problems and decrease shame and self-stigmatisation.

## Clinical significance

- A group-based CFT approach can provide peer support and a sense of belonging for adolescent girls who have mental health problems.
- There is a need for interventions that help empower adolescents who have mental health problems and to give them hope for the future.
